# Study of Estrogen Receptor Alpha Gene Polymorphisms (*ERα*, *ESR1*) in Women with Ovarian Cancer

**DOI:** 10.3390/ijms27073239

**Published:** 2026-04-02

**Authors:** Honorata Łukasiewicz, Dariusz Samulak, Hanna Romanowicz, Beata Smolarz

**Affiliations:** 1 Department of Nursing, Faculty of Medicine and Health Sciences, The President Stanisław Wojciechowski Calisia University, 62-800 Kalisz, Poland; honorata.lukasiewicz@wp.pl; 2 Department of Obstetrics and Gynecology and Gynecological Oncology, Regional Hospital in Kalisz, 62-800 Kalisz, Poland; samulakd@wp.pl; 3 Department of Obstetrics, The President Stanisław Wojciechowski Calisia University, 62-800 Kalisz, Poland; 4 Laboratory of Cancer Genetics, Department of Pathology, Polish Mother’s Memorial Hospital Research Institute, Rzgowska 281/289, 93-338 Lodz, Poland; hanna-romanowicz@wp.pl

**Keywords:** ovarian cancer, *ESR1*, polymorphism, gene

## Abstract

Despite the growing knowledge about ovarian cancer, it has not yet been possible to develop an effective screening test for this cancer. Therefore, it seems necessary to identify new risk factors, such as genetic polymorphisms. The aim of this study was to demonstrate whether polymorphisms of the *ESR1* gene rs2234693 and rs9340799 may be involved in the development of ovarian cancer. The material for the study was DNA obtained from 100 ovarian cancer patients and 100 control groups. Polymorphisms were determined using the PCR-RFLP technique. The presence of the CC genotype (rs2234693) has been shown to more than double the risk of ovarian cancer (OR 2.21; *p* = 0.041). In the case of the second polymorphism, rs9340799, the carrier of the GG genotype more than doubles the risk of ovarian cancer (OR 2.62 *p* = 0.031). Analysis of *ESR1* gene haplotypes in relation to the rs2234693 and rs9340799 polymorphisms showed that the occurrence of TCAG and CCGG systems may be associated with a significant increase in the risk of ovarian cancer (OR 1.98, *p* = 0.043 and OR 2.45, *p* = 0.041, respectively). In the group of patients with ovarian cancer, a correlation was shown between the polymorphisms rs2234693 and rs9340799 in the tissues of ovarian cancers with the lowest stage compared to more advanced ovarian cancers, which may indicate a relationship between these factors and the stage of the tumor. Women’s age had no effect on the prevalence of individual genotypes or the associated risk of disease. Polymorphisms rs2234693 and rs9340799 of the *ESR1* gene may be associated with the occurrence of ovarian cancer.

## 1. Introduction

Recent data indicates that the global incidence of ovarian cancer is higher than 240,000 cases annually. The highest incidence rates are consistently observed in Europe, North America, Australia, and New Zealand, with particularly high rates in Central and Eastern Europe [1,2].

Ovarian cancer is generally considered the second most common gynecologic cancer, following endometrial (uterine) cancer. Most cases of ovarian cancer occur after menopause, predominantly affecting women aged 55–70, with the peak incidence occurring between the ages of 55 and 59 [3]. Consequently, about 70% of these cases are detected at an advanced stage (FIGO III–IV) (The International Federation of Gynecology and Obstetrics) [4].

Ovarian cancer is one of the most dangerous cancers affecting women. This is the result of a too late diagnosis. Therefore, research is being conducted to learn about new predictive markers for this disease. Research focuses on estrogen receptors.

Two subtypes of estrogen receptors: alpha (ER-α) (encoded by the *ESR1* gene located on chromosome 6q25.1) and beta (ER-β) (encoded by the *ESR2* gene located on chromosome 14q23) belong to the nuclear receptor family [5,6].

As a result of the analysis of the occurrence of estrogen receptors in different histopathological types of ovarian cancer, it was shown that the expression of estrogen receptors alpha and beta in ovarian cancer cells is dependent on the histopathological type [7].

The highest expression of ER-α is shown by endometrioid and serous tumors, while the clear cell type is devoid of it. The ER-alpha receptor is present in all types of ovarian cancers, with the highest incidence in the endometrioid type [8]. A change in the expression profile—especially the loss of ER-β—is considered a significant event leading to the development of ovarian cancer. An increase in the ratio of ER-apha to ER-beta due to an increase in ER-alpha and a decrease in ER-beta is considered a marker of ovarian carcinogenesis. High ER-beta expression in ovarian tumors correlates with better progression-free survival [9,10].

Ovarian cancer cell line studies confirm that estrogen receptors alpha and beta have different functions in regulating gene expression, including the progesterone receptor (PR) [11,12,13]. ER-α and ER-β receptors affect the activity of cyclin D1 (a protein of the cell cycle) in an oppositional way [14]. ER-α increases the expression of the cyclin D1 gene (*CCND1*)*,* which promotes cell development [15]. However, it does not directly affect the proliferation and motility of ovarian cancer cells [16]. ER-β has an inhibitory effect on cancer progression—it reduces the expression of the cyclin D1 gene [15], inhibits proliferation, limits the motility of ovarian cancer cells, and has a pro-apoptotic effect [16]. ER-β is an important regulator of cellular processes, and the loss of its expression may be a key event leading to the development of ovarian cancer [16].

Hormone therapy is considered as a therapeutic option in both types of serum ovarian cancer (low-grade and high-grade), with a higher probability of a positive response in the low-grade type (due to higher receptor expression) [17].

The fundamental clinical significance of ESR1 estrogen receptor gene polymorphisms results from their role as predictive and prognostic factors in hormone sensitive diseases. Modern genetic research often focuses on the analysis of polymorphisms of the ESR1 gene, in particular the PvuII (rs2234693) and XbaI (rs9340799) variants, which are located in the first intron of this gene. These variants are being studied for their relationship to various pathological and physiological conditions. Polymorphisms are studied in endometrial diseases [18], osteoporosis [19,20], prostate [21], breast [22,23,24] and ovarian cancer [25].

Studies have shown a significant association of the polymorphisms rs9340799 and rs2234693 with an increased risk of ovarian cancer [26,27]. Weiderpass et al. reported that α receptor gene polymorphism in ovarian cancer may affect cell proliferation and anti-apoptosis, so that it may affect a person’s susceptibility to ovarian cancer [28]. Bardin et al. showed that the increase in *ESR1*/*ESR2* mRNA ratio observed in ovarian cancer showed a decrease in the selective expression of *ESR1* and *ESR2* mRNA levels without significant changes. The study also found that there were no significant differences in *ESR1* mRNA levels in normal ovaries, benign tumors, and ovarian cancers [29]. A study by Doherty et al. showed that the T allele rs2295190 or another allele in an imbalanced state was associated with an increased risk of invasive ovarian cancer [30].

The research conducted in this paper concerned the importance of single nucleotide polymorphisms of the *ESR1* gene as a risk factor for ovarian cancer. The research is important due to the fact that at the moment there is no method that would meet the conditions of screening for ovarian cancer. Therefore, it is extremely important to separate patients from “high-risk” groups and to control them closely.

The research hypothesis regarding estrogen receptor alpha (*ESR1*, *ERα*) gene polymorphisms in ovarian cancer assumes that specific genetic variants modify tissue sensitivity to estrogens, which may lead to uncontrolled proliferation of ovarian epithelial cells and increase the risk of cancer development. The main assumptions of this hypothesis are based on the role of estrogens as key growth stimulators in hormonally dependent tissues.

## 2. Results

Hardy–Weinberg equilibrium was preserved in *ESR1* rs2234693 and rs9340799 polymorphisms in all of the examined female patients (*p* > 0.050). Polymorphisms were observed in the linkage disequilibrium, found between the analyzed groups of women (*p* ≤ 0.050).

Among the women (100) with ovarian cancer, 24 (24%) people had TT genotype, 51 (51%) had TC genotype, and 25 (25%) had CC (Table 1).

In this group, the frequency of alleles was, respectively: allele T-99 (49.5%) and allele C-101 (50.5%). In the control group, TT genotype was found in 36 cases (36%), TC in 47 cases (47%), and CC in 17 cases (17%). The allele distribution was as follows—allele T 59.5%, and allele C-40.5%. The CC genotype was statistically significantly more common in the study group compared to the control (*p* = 0.041).

Among the women (100) with ovarian cancer, 42 (42%) people had the AA genotype, 40 (40%) had the AG genotype, and 18 (18%) had the GG genotype (Table 2).

In this group, the frequency of alleles was, respectively: allele A-62 (62%), allele G-38 (38%). In the control group, genotype AA was found in 49 cases (49%), AG in 43 cases (43%), and GG in 8 cases (8%).

The distribution of alleles was as follows—allele A 70.5% and allele G-29.5%. The GG genotype was statistically significantly more common in the study group compared to the control (*p* = 0.031).

The results obtained allowed us to conclude that the presence of the CC genotype (rs2234693) more than doubles the risk of ovarian cancer OR (95% PU)—2.21 (0.99–4.92), *p* = 0.013. Female age had no effect on the prevalence of individual genotypes or the associated risk of disease (Table 3 and Table 4). The results of the analysis are also presented in the form of adjusted odds ratio.

In the case of the second of the studied polymorphisms—rs9340799, it was found, analogously to previous studies, that the presence of GG genotype more than doubles the risk of ovarian cancer OR (95% PU)—2.62 (1.03–6.64), *p* = 0.031 (Table 5), which was confirmed by the analysis of the recessive model OR (95% PU)—2.52 (1.04–6.11), *p* = 0.028 (Table 6).

Analysis of *ESR1* gene haplotypes in relation to the rs2234693 and rs9340799 polymorphisms showed that among the subjects, the occurrence of the TCAG system (OR (95% CI)—1.98 (0.97–4.04), *p* = 0.043), and especially CCGG (OR (95% CI)—2.45 (0.98–6.10), *p* = 0.041), to a statistically significant degree, may be associated with a significant increase in the risk of cancer in women. Detailed results of the analyses are presented in Table 7.

The CC genotype in G1 patients had a statistically significantly higher incidence rate (42%), while TT homozygous had a statistically significantly lower incidence rate (20%) compared to G2 + G3 women (C/C-13%, TT-27%). The C allele in G1 patients had a statistically significantly higher incidence rate (61%), while the T allele had a statistically significantly lower incidence rate (39%) compared to the G2 + G3 group (T-57%, C-43%). A statistically significant increase in the incidence of CC homozygous (OR 3.54; 95% CI 1.08–11.55, *p* = 0.031) was observed in patients in Grade I, according to the FIGO classification. In turn, in the case of the rs9340799 polymorphism, a statistically significant increase in the incidence of GG homozygous (OR 3.5; 95% CI 1.10–11.08, and *p* = 0.029) was observed in patients with tumor size < 5 cm. The GG genotype in G1 patients had a statistically significantly higher incidence rate (30%) compared to the G2 + G3 group of women (GG-10%). The G allele in G1 patients had a statistically significantly higher incidence rate (47%), while the A allele had a statistically significantly lower incidence rate (53%) compared to the G2 + G3 group (G-32%, A-68%). The results of the above analyses are summarized in Table 8 and Table 9.

## 3. Discussion

Global epidemiological reports indicate a steady increase in the tendency to develop ovarian cancer. Ovarian cancer, despite great efforts to detect it early, is a difficult problem in gynecology oncology. The symptoms of ovarian cancer are non-specific and very poorly expressed, while its symptoms are often mistakenly identified with ailments of the digestive system. At present, there are no reliable diagnostic methods enabling early diagnosis of ovarian cancer [31,32,33]. Ovarian cancer is diagnosed late, in advanced stages of the disease, because the first uncharacteristic symptoms are often ignored by patients. Despite the growing knowledge about ovarian cancer, it has not yet been possible to develop an effective screening test for this cancer [34]. Therefore, it seems necessary to identify new risk factors. It becomes justified to search for new molecular markers in order to be able to detect less advanced tumors to a greater extent and to apply treatments to them that give the best prognosis.

Ovarian cancer is a hormone-dependent disease, driven specifically by ovarian steroid hormones, such as estrogens [35,36,37,38,39]. During the follicular phase, these estrogens stimulate the division of epithelial cells and fibroblasts. Long-term, excessive exposure to estrogen, particularly without the opposing anti proliferative effects of progesterone, is a recognized factor in the development of hormone-dependent cancers [7,40,41,42]. Research indicates that in many cases of epithelial ovarian cancer, there is an over-expression of ERα. While some studies show up to 80% of ovarian cancers are ER-positive, approximately 60% of cases exhibit significantly high levels of ERα [43,44,45,46,47].

ERα is encoded by the ESR1 gene. This gene is known for its polymorphism, which means that it occurs in different variants (polymorphisms) in a population [48,49,50,51]. Studies show that PvuII-XbaI polymorphisms are strongly coupled to VNTR polymorphisms (minisatellite sequences, e.g., TA repeats) in the promoter region, which together form complex haplotypes that affect gene activity [52].

The rs2234693 (PvuII) polymorphism is found in the first base pair of introns with a length of 397 base pairs (bp), above exon 2 [52,53]. The substitution of cytosine (C) by thymine (T) is recognized by the restriction enzyme PvuII [52,53]. The rs9340799 (XbaI) polymorphism is located at a distance of 50 bp from the PvuII polymorphism site [54]. The substitution of guanine (G) by adenine (A) is recognized by the restriction enzyme XbaI [54]. The C allele is part of the functional binding site of the transcription factor B-myb, which leads to an increase (enhancement) of estrogen receptor transcription [55]. Patients with the CC genotype have higher ER-α expression and worse survival, while the presence of the T allele is associated with lower expression and better survival [55].

The PvuII polymorphism affects ESR1 mRNA splicing, which causes a change in the synthesis of the ESR1 protein [56]. Possible functional mechanisms attributed to XbaI polymorphism include altering the expression of the ERα gene by altering the binding of transcription factors and influencing the alternative splicing of this gene. It is hypothesized that the transcription factor B-myb may affect the function of XbaI [57].

Intronic SNPs in the *ESR1* gene, particularly those in intron 1, act as functional variants that increase the binding affinity for the B-myb transcription factor. This increased binding disrupts the normal, highly repressed state of the *ESR1* gene in non-hormone-dependent tissues, forcing over-expression of the estrogen receptor alpha protein, a key driver of ovarian carcinogenesis. B-myb works with the MuvB complex to form the MMB (Myb-MuvB) complex, which drives the expression of genes necessary for cell cycle progression (mitosis). Intronic SNPs (often in linkage disequilibrium with known risk variants) create novel or enhance existing binding motifs for the B-myb/MMB complex. When B-myb binds to these intronic locations in *ESR1*, it acts as a strong transcriptional enhancer. This leads to increased, constitutive expression of *ESR1* mRNA, overriding natural, tissue-specific suppression [58].

Beyond increasing the transcription rate, the binding of transcription factors like B-myb to intronic regions can interact with the splicing machinery. Studies suggest that these variants can influence the structure and stability of the *ESR1* transcript, potentially leading to increased synthesis of more stable or more actively translated ERα mRNA isoforms [59,60].

The high ERα levels, often coupled with local estrogen production (e.g., in endometrioid or serous ovarian cancers), lead to uncontrolled proliferation, bypassing normal apoptosis signals. In tissues that typically have low ERα expression, this unnatural elevation allows the tumor to exploit estrogen signaling, promoting cell growth and survival. B-myb is a known oncogene, and its high expression—associated with 55% of HGSOC (High-Grade Serous Ovarian Cancer) cases—disrupts the DREAM complex (a transcriptional repressor), further allowing unchecked cell cycle progression [61].

The B-myb-mediated enhancement of *ESR1* expression via intronic SNPs serves as a mechanism to hijack cellular proliferation pathways, promoting ovarian cancer growth and potentially influencing resistance to therapies.

Polymorphisms rs9340799 (XbaI) and rs2234693 (PvuII) are often analyzed together due to a strong linkage disequilibrium (LD), which means that they are inherited together more often than the case would suggest.

There are association studies that have shown an increased risk of breast cancer [62,63,64], osteoporosis [65,66], as well as a reduced risk of endometriosis [67] in the context of the rs9340799 and rs2234693 polymorphisms. Current research work focusing on ESR1 polymorphisms, in particular rs2234693 and rs9340799 and other, less frequently studied ones (such as rs746432, rs2077647, and rs532010) aims to assess their effect on the risk of developing hormone-dependent cancers such as endometrial cancer [7], breast cancer [68,69,70] or ovarian cancer [71].

The study analyzed the prevalence of polymorphic variants rs2234693 and rs9340799 of the *ESR1* gene in patients with ovarian cancer in correlation with clinical and pathomorphological features. The results suggest that the presence of the CC genotype (rs2234693) may increase the risk of ovarian cancer, while the presence of the TT genotype (rs2234693) may reduce the risk of developing this type of cancer.

In the case of the second polymorphism, rs9340799, the presence of the GG genotype more than doubles the risk of ovarian cancer. Analysis of ESR1 gene haplotypes in relation to the rs2234693 and rs9340799 polymorphisms showed that the occurrence of the TCAG system, and especially CCGG, may be associated with a significant increase in the risk of ovarian cancer. In the case of the rs2234693 polymorphism, the presence of the CC genotype (presence of the C allele) is associated with the stage of the tumor and is associated with the degree of its differentiation. In the case of the rs9340799 polymorphism, the presence of the GG genotype (presence of the G allele) is associated with the size of the tumor and the degree of anaplasia. Women’s age had no effect on the prevalence of individual genotypes or the associated risk of disease.

Our results are in line with research conducted by Indian scientists. Research in India suggests that variants of the *ESR1* gene may be linked to the etiology of ovarian cancer in this ethnic group. Some analyses indicate a significant association of rs2234693 genotypes with susceptibility to reproductive diseases in women in India [72]. A significant increase in the incidence of the C allele was observed in Indian patients with ovarian cancer, similarly to Polish women with ovarian cancer [71]. The results are interesting from a demographic point of view.

The Polish population is relatively homogeneous in terms of genetics compared to other regions of the world. The Hindu population is known for its very high genetic diversity, resulting from historical caste isolation, which leads to the occurrence of specific polymorphisms within individual groups. The study was conducted on a group of Indian patients (n = 180) in a similar number to the Polish group [70]. A similar methodology (PCR-RLFP) was used.

The results consistent with those presented in the paper were also obtained by the researchers of the Pemmaraju et al. [25]. In this case, the PCR-RFLP technique was used, as in the Polish studies. In the case of the rs9340799 polymorphism, they showed a predominance of the presence of the AG genotype (70%) in patients with ovarian cancer. Contrary to the presented work, the distribution of the rs9340799 and rs2234693 haplotypes did not show any significant differences. The majority of ovarian cancer patients studied were carriers of the G allele, while control patients were carriers of the A allele. AG and GG genotypes may be a risk factor for ovarian cancer. However, the study included a smaller study group (40 patients). Therefore, the results obtained should be treated with caution as preliminary.

Based on existing scientific literature, your pilot study findings regarding the potential association of ESR1 polymorphisms rs2234693 and rs9340799 with ovarian cancer are supported by specific studies, although the overall understanding of this association remains limited and sometimes inconsistent [27,30,66,71].

A 2011 study on Indonesian women found that the heterozygous genotype TC of ESR1 rs2234693 (PvuII) and the heterozygous AG genotype of ESR1 rs9340799 (XbaI) were more frequent in patients with epithelial ovarian cancer than in controls, suggesting they may play a role in the etiology of the disease [27]. Because Primary Ovarian Insufficiency (POI) is linked with premature ovarian loss, studies on ESR1 rs2234693 and rs9340799 are highly relevant to ovarian dysfunction. Studies indicate a strong association between these polymorphisms and POI risk in Iranian and Korean populations [71,72].

Studies suggest that specific combinations (haplotypes) of rs2234693 and rs9340799, rather than individual SNPs, may confer a higher risk for gynecological dysfunction, with certain genotypes being more prevalent in diseased populations [27,30,66,71,72,73,74].

Similar to findings in breast cancer, studies on ESR1 polymorphisms in gynecological cancers often show conflicting results across different ethnic populations. While ESR1 is recognized as a major mediator of estrogen action (and thus relevant to ovarian cancer, a hormone-related cancer), many large consortium studies have historically focused on other polymorphisms, such as ESR1 rs2295190. As indicated in our study, the literature on the specific functional consequences of rs2234693 and rs9340799 in ovarian cancer is limited, necessitating further studies to confirm their role as markers of disease susceptibility.

Epidemiological studies have shown no significant association between *ESR1* PvuII polymorphism and overall cancer risk in homozygous (TT vs. CC) and heterozygous (TT vs. CT) models. A statistically significant relationship was observed only in the T vs. C allele model. The results suggest that the T genotype may reduce the risk of certain cancers: prostate cancer, leiomyomas, hepatocellular carcinoma. ESR1 PvuII (rs2234693 T>C) polymorphism appears to have only a minor effect on tumor susceptibility [75]. In the future, large-scale epidemiological studies are warranted to verify these results.

In the work of the team of Kutilin et al., genes regulating apoptosis, DNA repair, cell proliferation, metabolism and estrogen regulation in tumor and normal cells of high- and low-grade serous adenocarcinoma of ovaries were analyzed [76]. Molecular characterization of serous ovarian adenocarcinoma was performed. Using qPCR, the relative number of copies of 34 genes in normal and cancerous ovarian cells (collected by laser microdissection from paraffin blocks from 200 patients) was examined [76]. The main molecular markers of serous ovarian adenocarcinoma were identified, which include changes in the number of copies of genes: *PIK3CA*, *BCL2*, *BAX*, *CASP3* and *CASP8*. Based on the variation in the number of gene copies, two main molecular subtypes were distinguished, correlating with histological classification:

- High malignancy (HGSC): Associated with *MDM2*, *SOX2, ESR1*, *CYP1B1*, *SULT1E1*, *TP53*, and *BRCA*2 genes.
- Low malignancy (LGSC): Associated with the genes *PIK3CA*, *PTEN*, *BCL2*, *BAX* and *CASP3*.

Further molecular heterogeneity was demonstrated—the subtype of high degree of malignancy was divided into 3 subgroups, and the low-malignant subtype into 4 subgroups. The study confirms the existence of significant molecular differences between low- and high-malignant serous adenocarcinoma, which is the basis for their different clinical course and broadens the knowledge of the mechanisms of ovarian carcinogenesis.

There are reports indicating that mutations in the *ESR1* gene may affect endocrine therapy, often translating into its resistance [77]. Mutations in the *ESR1* gene (Y537S the most common, often in combination with others, D538G, and mutations in the L536 position, e.g., L536R/H/P/Q) are an important mechanism of acquired resistance to aromatase inhibitors (IAs) in hormone-dependent cancers. These mutations lead to the formation of a constitutively active estrogen receptor. This means that the receptor works without the presence of estrogen, which makes cancer cells hormone-independent and thus resistant to aromatase inhibitors that lower estradiol levels. Cancers with *ESR1* mutations have been shown to exhibit higher *ESR1* mRNA expression and activation of estrogen-dependent pathways, which drives disease progression. These mutations most often do not occur in primary tumors but appear during long-term therapy with aromatase inhibitors [77].

Confirmed evidence indicates that endometriosis, especially in its advanced forms, significantly increases the risk of developing certain types of ovarian cancer. Women with endometriosis have more than a four times higher risk of developing ovarian cancer compared to women without the disease. In the case of deeply infiltrating endometriosis and ovarian endometriosis (chocolate cysts), this risk increases almost ten times [78].

The latest study by researchers from the United States analyzed data on almost 500 thousand women in the state of Utah between the ages of 18 and 55. The study confirmed that the overall risk of developing ovarian cancer is 4.2 times higher in women with endometriosis compared to healthy women. The risk of type I ovarian cancer is 7.48 times higher. This group includes cancers that tend to grow more slowly, such as endometrioid, clear cell or mucinous cancer. The risk of type II ovarian cancer is 2.7 times higher. These are more aggressive and more difficult to treat, including serous carcinoma with a high degree of malignancy. In women with deep-infiltrating endometriosis or chocolate cysts (endometrioma), the overall risk of ovarian cancer was almost ten times higher (9.66), and in the case of type I cancer it increased as much as 19 times [79].

Literature data indicate that an increase in estradiol levels and ER-β-β expression, polymorphic genotypes and alleles of the ERβ rs4986938 G/A gene, and the frequency of ERα rs9340799 A/G alleles play a role in endometriosis [67].

A meta-analysis of 18 studies (including 4975 patients: 2222 in the endometriosis group and 2753 in the control group) found no significant association between the ESR1 Pvull (C>T) and Xbal (A>G) polymorphisms and the overall risk of developing endometriosis. Subgroup analysis indicated the existence of a weak relationship between PvuII polymorphism and endometriosis only in stages I–III (mild/medium) and only in the recessive model. The association of XbaI polymorphism with endometriosis was limited only to the non-PCR-RFLP genotyping method (other than standard PCR-RFLP) and also occurred only in the recessive model. PvuII and XbaI polymorphisms in the *ESR1* gene are not strong risk factors for endometriosis, and the suggested associations concern only specific subgroups (disease stage, research method, genetic model) [80].

According to our pilot study, polymorphisms of the *ESR1* gene, rs2234693 and rs9340799, may be associated with ovarian cancer. The current state of knowledge about *ESR1* polymorphisms in ovarian cancer remains limited. Therefore, further research is necessary and justified to explore this topic.

### Limitations

The sample size of 100 cases and 100 controls is very small for a genetic association study and represents a major limitation, as such studies often require hundreds or thousands of subjects to achieve sufficient statistical power (e.g., 80% power) to detect moderate effects. Small studies are often only capable of detecting very large genetic effects, missing more common, modest associations. With low power, the study may fail to detect a true genetic association, leading to the conclusion that no relationship exists when it actually does. Smaller samples are more sensitive to random variations and noise, which can produce spurious, statistically significant results (the “winner’s curse” or overestimation of effect sizes). Findings from a small sample are less likely to represent the broader population.

The control group consisting of women undergoing laparoscopy for non-malignant diseases is not a standard. Healthy control population is a valid and significant point in clinical study design. Women undergoing laparoscopy for gynecological issues are not truly “normal.” Conditions such as endometriosis or benign ovarian cysts can share inflammatory or genetic pathways with malignant ovarian tumors. These underlying conditions could be associated with the same genetic markers being investigated for cancer, leading to an underestimation of the true genetic difference between the study group and the general population. Research indicates that “normal” tissue in the vicinity of endometriosis can exhibit different molecular, hormonal, and epigenetic profiles compared to truly healthy tissue. While laparoscopy is a standard diagnostic tool for benign, non-malignant gynecological conditions, it does not completely guarantee that the surrounding tissues are free from pre-cancerous, molecularly, or genetically abnormal changes, particularly in studies focused on subtle genetic marker. It is worth noting that collecting ovarian tissue from completely healthy women in the general population is ethically difficult and rarely seen in invasive studies, which often forces researchers to use “hospital control”. The future studies should utilize healthy, age-matched, non-gynecological patients (or tissue banks) to validate the molecular finding. The results are preliminary and require replication on much larger cohorts (e.g., from genomic consortia).

## 4. Materials and Methods

**Figure 1:**
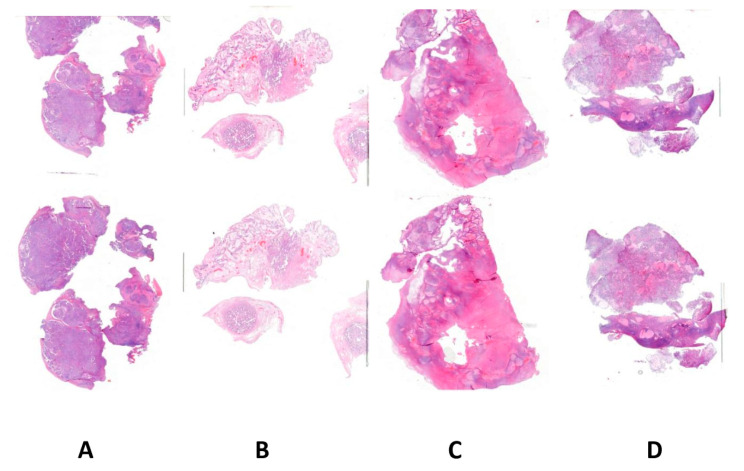
(**A**) Papillary serous right ovarian cancer G-2. Invasion of blood vessels and infiltration of the capsule are visible. (**B**) Endometrioid adenocarcinoma of the left ovary. (**C**) Clear cell ovarian cancer. (**D**) Clear cell ovarian cancer (haematoxylin-eosin staining). (from Department of Pathology, Polish Mother’s Memorial Hospital Research Institute, Lodz, Poland). Image obtained from the scanner (Case Viewer 2.3, 3D Histech, Budapest, Hungary); magnification 200×.

The study involved 100 patients with histologically confirmed ovarian cancer diagnosis. Archival material in the form of paraffin blocks containing sections from cancerous ovarian tumors was used for the research. All diagnosed tumors were classified according to the criteria of the International Federation of Gynecology and Obstetrics (FIGO). Histological typing and classification were performed in accordance with the WHO classification. Histological examinations were carried out by experienced pathologists using a digital scanner of specimens and software for viewing them (Case Viewer 2.3, 3DHistech, Budapest, Hungary). Histological specimens were scanned using a Panoramic scanner (3DHistech, Budapest, Hungary) to obtain digital images (Figure 1).

Normal ovarian tissue was taken from women undergoing laparoscopy for non-malignant diseases. To ensure that the selected histological material is representative of both cancerous and non-cancerous tissue, each tissue sample, qualified for DNA extraction, was initially checked by a pathologist. DNA from normal ovarian tissue (n = 100) served as a control. The control group consisted of unrelated people, without chronic diseases and without a history of ovarian or other cancers. From paraffin blocks, 2 pieces of 5 μm-thick were taken into 2 mL of Eppendorf tubes. Histopathological and genetic tests were carried out at the Department of Clinical Pathomorphology of the Polish Mothers Memorial Hospital Research Institute (PMMH-RI) and the Laboratory of Cancer Genetics of the Department of Clinical Pathomorphology of the PMMH-RI. The research was approved by the Bioethics Committee No. 34/2025 at the Polish Mothers Memorial Hospital Research Institute in Lodz. The characteristics of patients are presented in Table 10.

### 4.1. DNA Isolation

DNA extraction from the material was performed using the commercially available QIAamp DNA FFPE Tissue Kit (Qiagen GmbH, Hilden, Germany), in accordance with the manufacturer’s instructions. The purity of the obtained DNA preparations was determined by spectrophotometric method by measuring the absorbance of each sample twice at wavelengths of 260 nm and 280 nm. The accepted criterion of DNA purity was the A260/A280 value within the range of 1.8–2.0. DNA concentration was determined by spectrophotometric method based on the absorbance value measured at a wavelength of 260 nm. This value corresponded to the following relationships:

1OD = 50 μg DNA/mL

### 4.2. Analysis of ESR1 Polymorphisms

Based on the data available in the National Center for Biotechnology Information (http://www.ncbi.nlm.nih.gov/snp, accessed on 26 May 2020) SNP database, two single nucleotide polymorphisms located in the *ESR1* gene were selected. The selected SNPs are NC_000006.11:g.152163335T>C (rs2234693) and NC_000006.11:g.152163381A>G (rs9340799). An analysis of the incidence of the above-mentioned polymorphisms in both ovarian cancer patients and controls was performed using PCR reaction combined with restriction analysis, PCR-RFLP (ang. Polymerase Chain Reaction-Restriction Fragments Length Polymorphism). Selected fragments of the *ESR1* gene were amplified and then digested using restriction enzymes—rs2234693 (*PvuII*) and rs9340799 (*XbaI*). The nucleotide sequences of the primers used, were as follows:

forward 5′-CTG CCA CCC TAT CTG TAT CTT TTC CTA TTC TCC-3′

reverse 5′-TCT TTC TCT GCC ACC CTG GCG TCG ATT ATC TGA-3′

The PCR reaction was performed at a final volume of 10 μL. The reaction mixture contained approximately 50 ng of genomic DNA, 1 μL of 5 μM solution of each primer, 1 μL of GeneAmp 10× PCR Buffer with MgCl_2_ (Applied Biosystems, Waltham, MA, USA), 0.2 μL of AmpliTaq Gold^®^ polymerase (5 U/μL) (Applied Biosystems, USA), 1 μL of dNTPs of 10 μM for each of the deoxyribonucleotides, and water. Initial denaturation was carried out for 5 min at 95 °C, then the selected fragment of the studied gene was amplified during 40 cycles with the following parameters: 30 s at 95 °C, 1 min at 62 °C and 1 min at 72 °C. Final elongation was carried out at 72 °C for 10 min.

The products obtained as a result of DNA amplification were subjected to digestion lasting 16 h, using commercially available restriction enzymes PvuII and XbaI. The reaction products were then electrophoresed in 2% agarose gel, stained with ethidium bromide and visualized in ultraviolet light.

The size of the products obtained in the PvuII polymorphism analysis (rs2234693, T/C) and in the XbaI polymorphism analysis (rs9340799, A/G) are listed in Table 11.

### 4.3. Statistical Analysis

The statistical analysis of the obtained results was carried out with the help of STATISTICA 11 (StatSoft, Cracow, Poland), Chaplin 1.2 (genetics.emory.edu) and THESIAS (www.genecanvas.org, accessed on 20 April 2020). The effect of *ESR1* gene polymorphisms on ovarian cancer risk was assessed using odds ratio (OR) and a 95% confidence interval (CI) that was determined using a logistic regression model. In this case, the OR allows you to assess how many times the risk of disease increases/decreases when the unit of the variable treated as a risk factor increases/decreases. In order to check whether the Hardy–Weinberg equilibrium law is maintained in the studied populations, the calculation program on the www.ihg.gfs.de website (Institute of Human Genetics, Technical University Munich and Helmholtz Center Munich, accessed on 1 January 2021) was used. To investigate the effect of *ERS1* gene haplotypes on the risk of cancer, the Chaplin 1.2 and THESIAS programs version 1.2.3 (accessed on 1 January 2019) were used.

All statistical tests were performed at a significance level of α = 0.05. The consistency of the distribution of values of the studied variables with the normal distribution was assessed using the Shapiro–Wilk test. In order to verify the hypothesis of age significance in the study and control groups, χ^2^ analysis was used. The result was considered statistically significant at a significance level *p* of less than 0.05. In the case of multiple testing (haplotypes, and subgroup analyses by FIGO stage, grade, and tumor size), Bonferroni correction was used.

## 5. Conclusions

Our pilot study aligns with literature suggesting that polymorphisms in the first intron of the *ESR1* gene (rs2234693/PvuII and rs9340799/XbaI) are relevant to ovarian dysfunction. Continued investigation into these variants is important to clarifying their exact contribution to ovarian cancer risk.

## Figures and Tables

**Table 1:** Genotype and allele frequency distributions of the *ESR1* gene polymorphism rs2234693 in the group of patients with ovarian cancer and in the control group.

	Patients (n = 100)		Control (n = 100)		OR ^a^ (95% CI) ^b^	*p*-Value ^c^
	Numbers	(%)	Numbers	(%)		
TT	24	24.0	36	36.0	1.00 Ref.	
TC	51	51.0	47	47.0	1.62 (0.84–3.12)	0.095
CC	25	25.0	17	17.0	2.21 (0.98–4.92)	0.041
T	99	49.5	119	59.5	1.00 Ref.	
C	101	50.5	81	40.5	1.49 (1.01–2.22)	0.028

^a^ OR, odds ratio; ^b^ 95% CI, confidence interval; ^c^ logistic regression model; *p* ≤ 0.050 is considered significant.

**Table 2:** Genotype and allele frequency distributions of the *ESR1* gene polymorphism rs9340799 in the group of patients with ovarian cancer and in the control group.

	Patients (n = 100)		Control (n = 100)		OR ^a^ (95% CI) ^b^	*p*-Value ^c^
	Numbers	(%)	Numbers	(%)		
AA	42	42.0	49	49.0	1.00 Ref.	
AG	40	40.0	43	43.0	1.08 (0.60–1.96)	0.453
GG	18	18.0	8	8.0	2.62 (1.04–6.64)	0.031
A	62	62.0	141	70.5	1.00 Ref.	
G	38	38.0	59	29.5	1.69 (1.01–2.83)	0.030

^a^ OR, odds ratio; ^b^ 95% CI, confidence interval; ^c^ logistic regression model; *p* ≤ 0.050 is considered significant.

**Table 3:** Distribution of genotypes of the rs2234693 polymorphism of the *ESR1* gene in the patients and control groups, and analysis of the risk of ovarian cancer.

SNP/Genotype rs2234693 (PvuII)	Patients (%)/Control (%)	OR (95% CI) ^a^	*p*	OR (95% CI) ^b^	*p*
TT	24 (24.0)/36 (36.0)	1.00 Ref.		1.00 Ref.	
TC	51 (51.0)/47 (47.0)	1.62 (0.84–3.12)	0.095	1.61 (0.81–3.02)	0.096
CC	25 (25.0)/17 (17.0)	2.21 (0.99–4.92)	0.013	2.20 (0.98–4.82)	0.010
p-trend ^c^	0.04				

^a^ raw ^b^ age adjusted. ^c^ additive genetic model (Cochran-Armitage test).

**Table 4:** Distribution of rs2234693 polymorphism genotypes of the *ESR1* gene in the patients and control groups—dominant and recessive model.

SNP/Genotype rs2234693 (PvuII)	Patients (%)/Control (%)	OR (95% CI) ^a^	*p*	OR (95% CI) ^b^	*p*
TC or CC vs. TT ^c^	76 (76.0)/64 (64.0)	0.57 (0.31–1.03)	0.044	0.55 (0.30–1.01)	0.044
TC or TT vs. CC ^d^	75(75.0)/83 (83.0)	1.62 (0.82–3.24)	0.112	1.61 (0.81–3.14)	0.102

^a^ raw ^b^ age adjusted. ^c^ dominant genetic model. ^d^ recessive genetic model.

**Table 5:** Distribution of rs9340799 gene polymorphism genotypes in the patients and control groups, and analysis of ovarian cancer risk.

SNP/Genotype rs9340799 (XbaI)	Patients (%)/Control (%)	OR (95% CI) ^a^	*p*	OR (95% CI) ^b^	*p*
AA	42 (42.0)/49 (49.0)	1.00 Ref.		1.00 Ref.	
AG	40 (40.0)/43 (43.0)	1.08 (0.59–1.97)	0.453	1.07 (0.57–1.95)	0.450
GG	18 (18.0)/8 (8.0)	2.62 (1.03–6.64)	0.031	2.60 (1.01–6.04)	0.030
p-trend ^c^	0.002				

^a^ raw ^b^ age adjusted. ^c^ additive genetic model (Cochran-Armitage test).

**Table 6:** Distribution of genotypes of the rs9340799 polymorphism of the *ESR1* gene in the patients and control groups—dominant and recessive model.

SNP/Genotype rs9340799 (XbaI)	Patients (%)/Control (%)	OR (95% CI) ^a^	*p*	OR (95% CI) ^b^	*p*
AG or GG vs. AA ^c^	58 (58.0)/51 (51.0)	0.75 (0.43–1.31)	0.197	0.75 (0.42–1.31)	0.195
AG or AA vs. GG ^d^	82 (82.0)/92 (92.0)	2.52 (1.04–6.11)	0.028	2.52 (1.04–6.10)	0.028

^a^ raw ^b^ age adjusted. ^c^ dominant genetic model. ^d^ recessive genetic model.

**Table 7:** *ESR1* gene haplotypes and ovarian cancer risk.

Haplotypes (rs2234693; rs9340799)	Patients (%)/Control (%)	OR (95% CI) ^a^	*p* ^b^/Bonferroni Correction
TT-AA	24 (24.0)/36 (36.0)	1.00 (Reference)	
TC-AA	17 (17.0)/14 (14.0)	1.82 (0.76–4.37)	0.130/0.067
TC-AG	37 (37.0)/28 (28.0)	1.98 (0.97–4.04)	0.043/0.021
CC-AG	4 (4.0)/9 (9.0)	0.54 (0.15–1.91)	0.259/0.139
CC-GG	18 (18.0)/11 (11.0)	2.45 (0.98–6.10)	0.041/0.021

^a^ raw, ^b^ *p* ≤ 0.050 is considered significant.

**Table 8:** Genotype and frequency allele distributions of polymorphism rs2234693 in ovarian cancer patients, depending on clinicopathological features.

	Tumor Size < 5 cm (n = 41)	Tumor Size > 5 cm (n = 59)	OR ^a^ (95% CI) ^b^	*p* ^c^/Bonferroni Correction
rs2234693	number (%)	number (%)		
TT	11 (26)	13 (22)	1.00 Ref	
TC	15 (37)	36 (61)	0.49 (0.18–1.34)	0.128/0.066
CC	15 (37)	10 (17)	1.77 (0.57–5.50)	0.240/0.128
T	37 (45)	62 (53)		
C	45 (55)	56 (47)	1.34 (0.77–2.37)	0.187/0.098
	FIGO I (n = 39)	FIGO II + III (n = 61)		
TT	9 (23)	15 (25)	1.00 Ref	
TC	13 (33)	38 (62)	0.57 (0.29–2.35)	0.465/0.268
CC	17 (44)	8 (13)	3.54 (1.08–11.5)	0.031/0.016
T	31 (40)	68 (56)	1.00 Ref	
C	47 (60)	54 (44)	1.91 (1.07–3.40)	0.019/0.009
	G1 (n = 40)	G2 + G3 (n = 60)		
TT	8 (20)	16 (27)	1.00 Ref	
TC	15 (38)	36 (60)	0.83 (0.16–1.45)	0.156/0.081
CC	17 (42)	8 (13)	4.25 (1.28–14.03)	0.015/0.008
T	31 (39)	68 (57)	1.00 Ref	
C	49 (61)	52 (43)	2.07 (1.17–3.68)	0.001/0.0005

^a^ OR, odds ratio; ^b^ 95% CI, confidence interval; ^c^ *p* ≤ 0.050 is considered significant.

**Table 9:** Genotype and frequency allele distributions of polymorphism rs9340799 in ovarian cancer patients, depending on clinicopathological features.

	Tumor Size < 5 cm (n = 41)	Tumor Size > 5 cm (n = 59)	OR ^a^ (95% CI) ^b^	*p* ^c^/Bonferroni Correction
rs9340799	number	number		
AA	13 (32)	29 (49)	1.00 Ref	
AG	17 (41)	23 (39)	1.64 (0.67–4.08)	0.196/0.103
GG	11 (27)	7 (12)	3.5 (1.10–11.08)	0.029/0.014
A	43 (52)	81 (68)		
G	39 (48)	37 (32)	1.98 (1.11–3.56)	0.015/0.007
	FIGO I (n = 39)	FIGO II + III (n = 61)		
AA	19 (49)	23 (38)	1.00 Ref	
AG	11 (28)	29 (48)	0.45 (0.18–1.15)	0.075/0.038
GG	9 (23)	9 (14)	1.21(0.40–3.66)	0.476/0.276
A	49 (63)	75 (61)	1.00 Ref	
G	29 (37)	47 (39)	0.94 (0.53–1.69)	0.484/0.281
	G1 (n = 40)	G2 + G3 (n = 60)		
AA	14 (35)	28 (47)	1.00 Ref	
AG	14 (35)	26 (43)	1.07 (0.43–2.68)	0.529/0.313
GG	12 (30)	6 (10)	4.0 (1.23–12.9)	0.017/0.008
A	42 (53)	82 (68)	1.00 Ref	
G	38 (47)	38 (32)	1.95 (1.09–3.50)	0.018/0.009

^a^ OR, odds ratio; ^b^ 95% CI, confidence interval; ^c^ *p* ≤ 0.050 is considered significant.

**Table 10:** Characteristics of ovarian cancer patients and controls.

	Patients (%)	Control Group (%)
group size	n = 100	n = 100
age (range)	35–72	37–69
age, average	52.11 ± 10.65	59.10 ± 10.11
<50 years	16 (16.0)	26 (26.0)
≥50 years	84 (84.0)	74 (74.0)
histological type		
serous cancer	40 (40.0)	
mucinous carcinoma	4 (4.0)	
endometrioid cancer	31 (31.0)	
clear cell carcinoma	2 (2.0)	
undifferentiated cancer	23 (23.0)	
G (grading)		
G1	40 (40.0)	
G2	18 (18.0)	
G3	42 (7.0)	
FIGO (staging)		
I	39 (39.0)	
II	13 (13.0)	
III	48 (48.0)	
tumor size		
>5 cm	41 (41.0)	
<5 cm	59 (59.0)	

**Table 11:** Size of products obtained in PvuII polymorphism analysis (rs2234693, T/C) and XbaI polymorphism analysis (rs9340799, A/G).

PvuII (rs2234693); base pairs	TT-1374 TC-374, 934, 440 CC-934, 440
XbaI (rs9340799); base pairs	AA-1374 AG-1374, 981, 393 GG-981, 393

## Data Availability

The original contributions presented in this study are included in the article. Further inquiries can be directed to the corresponding author.
